# From policy to practice: Lessons learned from an open science funding initiative

**DOI:** 10.1371/journal.pcbi.1011626

**Published:** 2023-12-07

**Authors:** Sonya B. Dumanis, Kristen Ratan, Souad McIntosh, Hetal V. Shah, Matt Lewis, Timothy H. Vines, Randy Schekman, Ekemini A. Riley

**Affiliations:** 1 Coalition for Aligning Science, Chevy Chase, Maryland, United States of America; 2 Aligning Science Across Parkinson’s (ASAP), Chevy Chase, Maryland, United States of America; 3 Strategies for Open Science (Stratos) and Incentivizing Collaborative Open Research (ICOR) Santa Cruz, California, United States of America; 4 DataSeer Research Data Services, Vancouver, British Columbia, Canada; 5 Howard Hughes Medical Institute, University of California, Berkeley, Berkeley, United States of America; University of Virginia, UNITED STATES

## Introduction

In the past few years, there has been a notable shift in the open science landscape as more countries and international agencies release recommendations and implementation guidelines for open scholarship [1–7]. In August 2022, the US White House Office of Science and Technology (OSTP) released a memo [8] with guidance that all federally funded research articles be (1) open access and (2) include sharing of underlying datasets in public repositories. The global open scholarship conversation has shifted from making a case for open science to developing operational workflows to assess, monitor, and enforce open policies that can normalize, simplify, and streamline these processes for use in daily research practice. As various workflows are proposed, there is a need for collective action across funders, institutions, and governments to align on open science policies and practices to reduce the cost and friction of adoption [9–12].

Here, we examine the practices of the Aligning Science Across Parkinson’s (ASAP) initiative [13], whose mission is to accelerate the pace of discovery and inform the path to a cure for Parkinson’s disease through collaboration, research-enabling resources, and data sharing [14,15]. ASAP was conceived through an *open-by-design* framework from the start. To learn more, please see the ASAP Blueprint for Collaborative Open Science [16], which provides a detailed overview of the ASAP open science policies, templates, and reports. Grantees within the ASAP Collaborative Research Network (CRN), an international, multidisciplinary, and multi-institutional network of collaborating investigators, are already required to be compliant with the recommendations of the OSTP memo by adhering to ASAP’s open science policies [17]. For example, ASAP requires the posting of a preprint at the time of (or before) article submission, immediate open access for all publications, and a mandatory CC-BY license. Additionally, at the time of publication, all underlying research outputs (protocols, code, datasets) must be posted to a FAIR repository [18,19] and all research outputs from ASAP-funded research must have DOIs or other appropriate identifiers, such as RRIDs for material resources, appropriately linked to the manuscript (see Table 1 for list of identifier types). Here, we evaluate the feasibility, ease, impact, and improvement to our open science policies as they were implemented within the ASAP CRN program and discuss our lessons learned to assist other funders and institutions considering open science implementation.

**Table 1:** Tracked research output types and identifier acronyms. This table highlights the different research outputs and the unique identifiers commonly ascribed to these output types as defined by Aligning Science Across Parkinsons.

Research output definitions	
**Dataset**	Data collection is an action where features of the real world (heights, reflectances, etc.) are captured by some device and become data points in a computer. A collection of data points about the same object or class of objects is a dataset. Reused data was generated in other publications or sources.
*Dataset categories that currently are not required to be included in the final dataset deposition*:	
*Quality control (QC) datasets*	Raw data from testing a sample of the output is not required, but measurements are recommended for inclusion to increase faith in the results presented. All data types can appear in this category.
*Confirmatory datasets*	Intermediate steps that produce the final dataset can be treated the same as QC datasets. Examples of confirmatory datasets include many assays, such as chromatography, genotyping, confirmatory sequencing, etc.
*Representative media datasets*	Visual and other datasets that sometimes require an enormous amount of space to store are not currently required for inclusion in repository uploads. Representative datasets can be new or reused datasets. Examples of these data types include image, video, and sound.
**Code**	Any usage of command-line software indicates that the authors must have custom scripts (which they wrote to operate the software), and these scripts need to be shared.
**Software**	Programs including software, packages, sites, databases, and other resources. New software is any program generated with ASAP funds. Appropriate citations are required for any use of preexisting software to analyze data or run simulations.
**Lab materials**	Stable resources include organisms, cell lines, plasmids, and antibodies that have registering bodies to mint RRIDs associated with lab material type.
**Protocols**	Standalone documents written recipe style that are archived/shared on a dedicated platform and referenced in subsections within the “Methods” of an article excluding lists and statistical analyses.
**Identifiers**	
**Accession number**	A unique number assigned by a particular database as a means of locating a specific output. Governments often use this to reference items in their database. For example, the National Center for Biotechnology Information assigns accession numbers to genetic data collections deposited there.
**DOI**	Digital object identifier is a type of identifier utilized to register digital objects such as manuscripts, datasets, protocols, and code.
**URL**	Uniform resource locator referencing the web address associated with a digital output.
**RRID**	Research Resource Identifier is a type of identifier utilized to register lab materials such as antibodies, cell lines, plasmids, etc. in a centralized database. RRIDs can also be used to register software that does not have an associated DOI.
**Kit number**	For specific protocols, companies will produce kits that bundle all the reagents required and provide detailed step-by-step instructions for the specific protocol. The kit number references the bundled lab materials and its associated batch.
**PID**	Persistent identifier is a long-lasting reference that links to the current address of the metadata of an output or the actual content. For example, DOI, accession numbers, and RRIDs are all considered a type of persistent identifier.

### Tracking compliance for open science

To assess ASAP’s effectiveness in upholding best open science practices for linking research outputs within an article and tracking compliance, ASAP partnered with an AI startup, DataSeer, which uses natural language processing and machine learning software to identify and assess the research outputs in a manuscript. The software simultaneously tallies the quantity, citations, and sharing status of newly generated and existing datasets, code, software, protocols, and lab materials. A DataSeer curator generates a report summarizing action items required for the article to meet compliance with ASAP policies. DataSeer receives articles through ASAP staff submissions and will return the resulting report assessment for ASAP staff to review and share with authors. Based on ASAP staff feedback, the authors may make amendments to update their manuscript. The submission, curation, and adjustment process are iterative, with the ASAP staff providing continual feedback to CRN teams until compliance is achieved. An example template of what the report looks like was deposited in Zenodo (https://doi.org/10.5281/zenodo.7504034).

**Fig 1:**
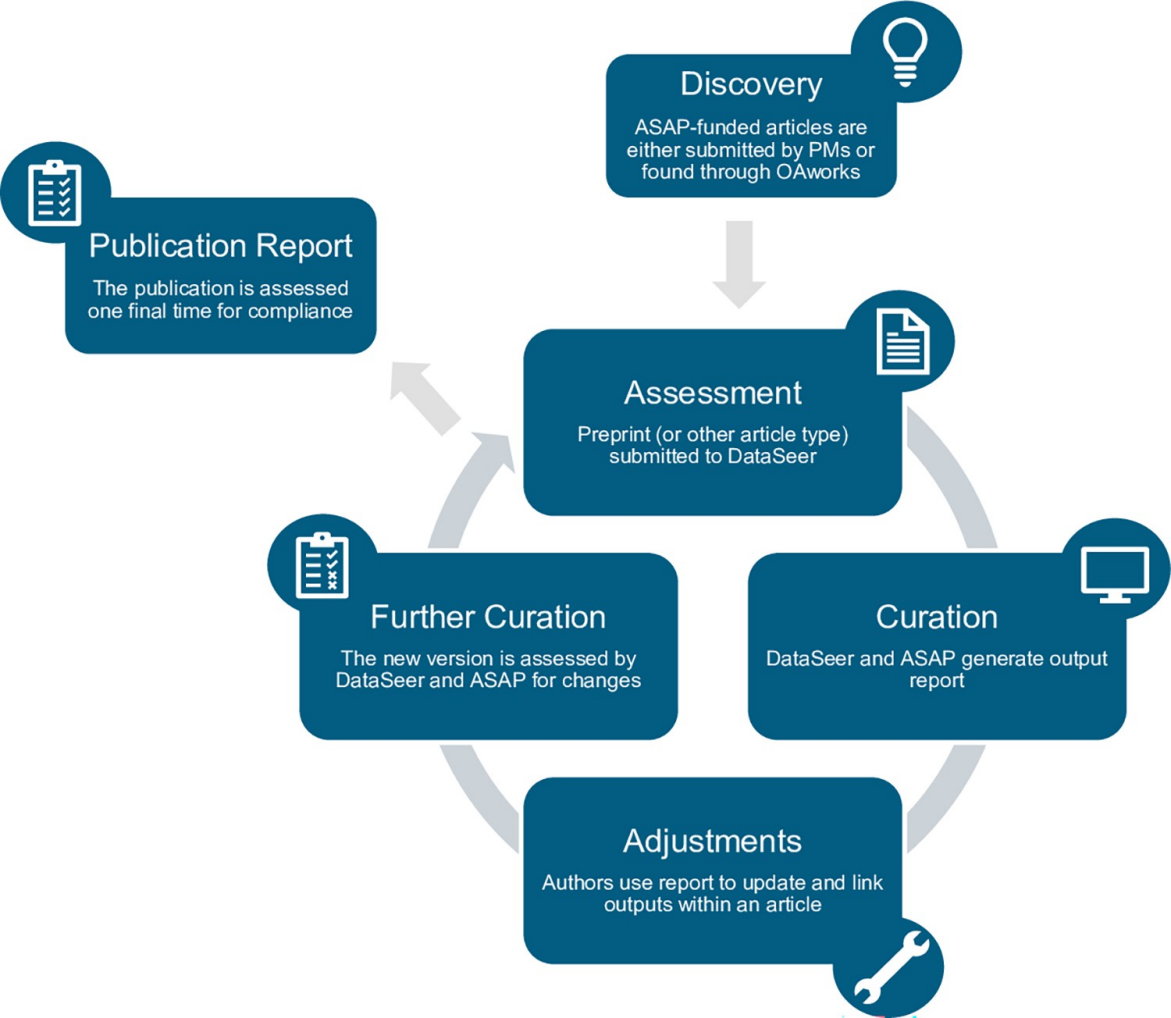
Schematic of the compliance workflow for ASAP grantees. Two main mechanisms discover ASAP-funded articles: either by team PMs or through OA.Report, and then submitted to the open science team on a rolling basis. OA.Report identifies papers by looking at the acknowledgment sections of preprints and publications for reference to Aligning Science Across Parkinson’s. Once received, DataSeer generates a compliance output report, which is checked by ASAP staff, and then shared with the article’s authors. Authors use the compliance report to understand what research outputs are not properly cited and recommendations for proper citation. After the article is revised, it is resubmitted to DataSeer and curated again. The assessment–curation–adjustment process can be repeated until all research outputs are appropriately cited. Finally, when the article is ready for publication, the report is assessed one final time for compliance.

ASAP currently supports 35 different teams within the CRN. A CRN team is led by a core group of 3 to 5 lead investigators, and together with their respective labs and other optional collaborators, they work to complete the goals of their grant. Most of the research articles submitted to ASAP staff for compliance review from the CRN teams come from the team’s project manager (PM), a position allocated within the CRN team’s grant budget. At the start of the award, teams must identify an interim PM within their lab and then hire a permanent full-time PM within 3 months of the award. The PMs assist with ASAP open science policy compliance, facilitate collaboration across the network through identifying synergistic opportunities and resources for their teams to leverage, support onboarding of new team members that join their CRN team, and ensure that teams are completing their listed deliverables within their grant. In addition to utilizing the PMs, ASAP staff also received a list of articles through OA.Report (RRID: SCR 023288). OA.Report has a discovery process in which they scrape the web for any mention of Aligning Science Across Parkinson’s within an article’s acknowledgment sections. See Fig 1 for the workflow schematic.

### Standardized research output compliance rules

Our initial challenge in running the reports and conducting this analysis was around establishing clear rules on what was considered an accurately cited research output. Currently, no community-wide accepted standards exist across output types. Therefore, ASAP and DataSeer developed criteria based on FAIR standards [18,19]. FAIR rules are well-established for data and generally applicable for code scripts but have yet to be concretely applied to other trackable research outputs in academic publications. We decided to count any output as properly shared if an output had a specific, functioning, stable identifier(s) associated with it. Table 1 lists the definitions of ASAP’s tracked research output types (data, code and software, lab resources, protocols) associated with a manuscript. Table 2 displays the criteria for how these output types would be considered appropriately shared per ASAP guidelines. Our current workflow is a foundational starting point that allows for future changes to the definitions to be implemented at scale.

**Table 2:** Identifier requirements for appropriately shared research outputs. This table highlights the different stable identifiers required for each specific output type to be counted as accurately cited in a manuscript.

	Data	Code and software	Lab materials	Protocols
**New**	URL or DOI	DOI (RRID if software)	RRID	URL or DOI
**Reused**	URL or DOI, if URL include date queried	Version, URL, RRID (if available)	Source, catalog/kit number, RRID (if available)	Citation, URL, DOI, or kit number

### Assessing impact: Compliance reports and measuring behavior change

Compliance reports from DataSeer track and evaluate baseline compliance, before any intervention, as well as compliance after an article is versioned. In the manuscript lifecycle, there can be multiple versions of a manuscript from the draft manuscript to the preprint posted, to the version submitted for peer review, to the resulting manuscript version released for publication. For this analysis, we tracked how the versions, drafts throughout the manuscript lifecycle, changed over time. This helps us understand the change over time as a research team amends any issues that ASAP staff raised related to output sharing in an article following a compliance report. To understand user behavior, we assessed output sharing in the first and second versions of the manuscripts through the DataSeer workflow (Fig 1). Because we continually refined the research output rules through February 2022, we restricted evaluation to articles submitted to DataSeer after March 1, 2022 (termed the first version). The subsequent submission (termed the second version) were articles submitted to DataSeer at least 2 business days ***after*** the first version. This criterion was included as sometimes a draft manuscript was received the day it was uploaded to a preprint server, which appeared online 2 days later. Within this criterion, 19 articles had a first and second version submission through the DataSeer system between March 1, 2022 and October 1, 2022, and these were the articles analyzed in our assessment.

**Fig 2:**
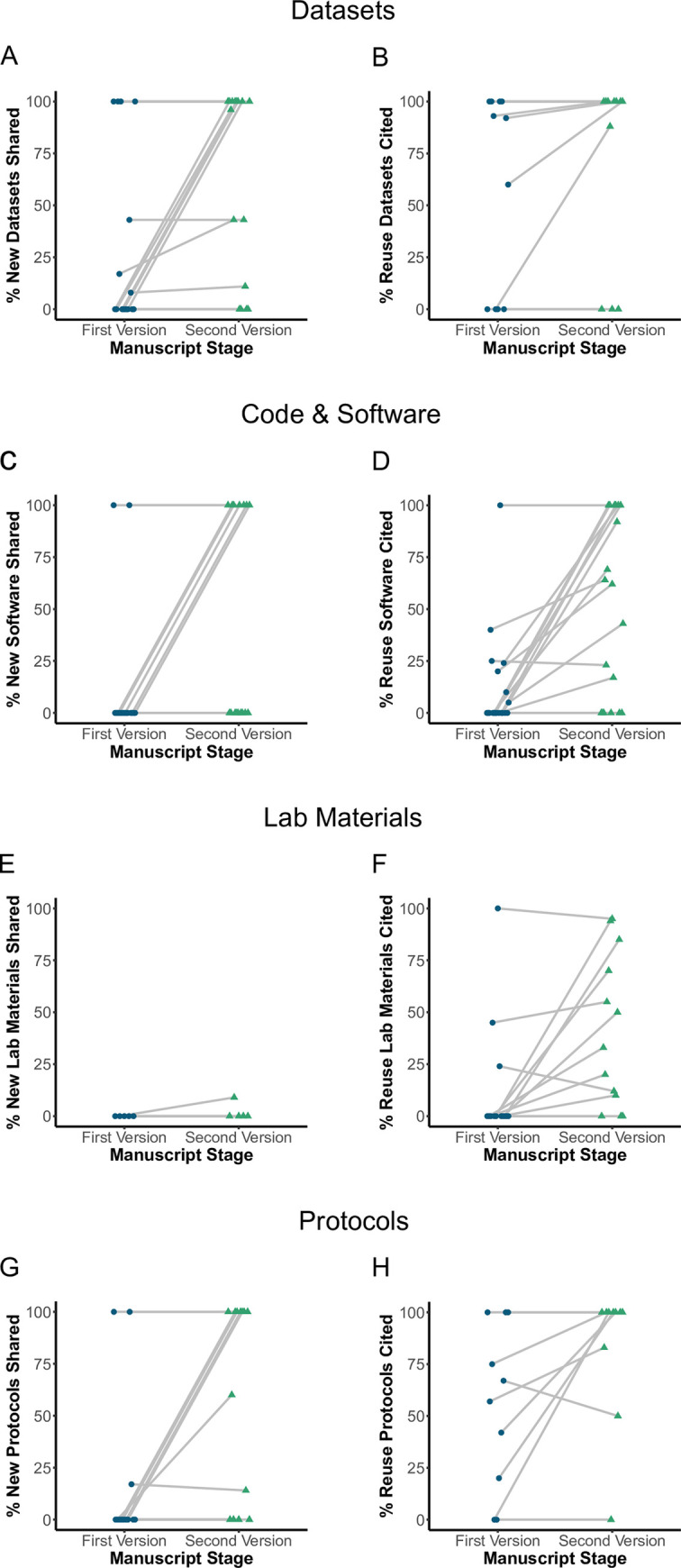
Compliance review increases the sharing of new and reuse research outputs. We assessed compliance with ASAP’s open science policy at 2 stages within the lifetime of ASAP-funded research articles: the first version submitted to ASAP for initial review and the subsequent version following said review. Compliance was assessed by measuring the percentage of novel and reuse research outputs (datasets, code/software, lab materials, and protocols) shared or cited in both versions. There were a total of 19 article pairs examined for this analysis. If a paper did not contain an output type in either the version 1 or version 2 assessment, it was excluded from the analysis for that output type. See Table 3 for the total number of papers included in each output assessment type. Within each panel, the y-axis represents the percentage of shared research outputs and the x-axis represents the 2 stages of manuscript development. Blue dots and green triangles represent unique manuscripts in the first and second versions. The first and second versions of research articles are connected via gray lines. **(A)** Percentage of new datasets shared across stages. **(B)** Percent reuse datasets cited across stages. **(C)** Percentage of new code software shared across stages. **(D)** Percentage of reuse code and software cited. **(E)** Percentage of new lab materials shared across stages. **(F)** Percentage of reuse lab materials cited across stages. **(G)** Percentage of new protocols shared across stages. **(H)** Percentage of reuse protocols cited across stages.

We tallied the number of outputs identified and cited appropriately in the first and second version of the manuscripts based on ASAP’s standards (Table 2) for each newly generated and reused research output type (datasets, code and software, lab materials, and protocols). In Table 3, we report the numbers in 2 ways: the proportion of outputs shared across all outputs identified in all papers (the purple columns) and the average proportion of outputs shared per paper (green columns). Please see Table 3 and Fig 2 for a summary of the overall results by output type.

**Table 3:** Summary statistics for assessing compliance over time. Here, we report the summary statistics around the proportion of outputs shared across all outputs identified from papers in version 1 of the manuscript, before our review, and version 2 of the manuscript, after we highlighted the changes that needed to be made in the manuscript to accurately cite all research outputs.

		Version 1	Version 2	Version 1	Version 2
	Number of papers referencing an output type	Total outputs shared across all papers/total outputs identified across all paper	Total outputs shared across all papers/total outputs identified shared across all papers	Average percentage shared per paper	Average percentage shared per paper
**New datasets**	16	13/108 (12%)	95/137 (69%)	29.25%	62.05%
**New code and software**	16	2/31 (6%)	21/32 (65%)	12.50%	43.75%
**New lab materials**	5	0/424 (0%)	28/428 (6%)	0.00%	1.81%
**New protocols**	13	12/89 (13%)	52/99 (53%)	16.67%	59.56%
**Reused datasets**	11	61/71 (86%)	74/78 (95%)	58.65%	71.59%
**Reused code and software**	19	21/209 (10%)	125/216 (58%)	11.74%	50.98%
**Reused lab materials**	13	48/281 (17%)	156/387 (40%)	13.02%	40.33%
**Reused protocols**	10	24/49 (49%)	49/52 (94%)	56.05%	83.33%

There were a total of 19 article pairs examined for this analysis. If a paper did not contain an output type in either the version 1 or version 2 assessment, it was excluded from the analysis for that output type.

As expected, all output types show an improvement in their sharing status in the second version of the manuscript after receiving a compliance report summarizing action items authors needed to take for each output type to achieve compliance per ASAP policies (Fig 2 and Table 3). In our follow up discussions with authors, we began to catalog the unique challenges by output type that prevented authors from achieving 100% compliance even after our outreach to authors. Below is a discussion of these specific barriers.

### Datasets

On average, sharing of newly generated datasets increased from 12% in the first version to 69% in the second version (Fig 2A and Table 3). When asked why all datasets were not shared for a specific manuscript, the most common response was that the ASAP grantee had not generated the dataset in question, and the grantee could not control the actions of collaborators who were not funded by ASAP. Rarely is a funder the sole funder of a research publication, and ASAP began to draft guidelines for approaching new research collaborations and discussing ASAP policies.

### Code and software

For the purposes of this discussion, we will use the term software liberally to apply to both code and software outputs. When assessing software sharing in the second version of manuscripts, there was a strong upward trend toward more software shared. Proper software linkages jumped from 6% in the first version to 65% in the second version of manuscripts for newly generated outputs. For software citing preexisting software (reused software citations), the numbers increased from 10% in the first version to 58% on average in the second version (Fig 2C and 2D). There were 2 factors often cited for noncompliance with properly citing software. First, there is a lack of consensus and education on how to cite software. There are 2 usual routes to citing software: a DOI generated by Zenodo, a general all-purpose open repository, or via a Research Resource Identifier (RRID). Most members of our network were unaware that Zenodo has capabilities to sync up with GitHub, a common web platform for writing code, to provide a digital object identifier (DOI) and make that code citable in a paper with an identifier. For those not using GitHub, they were unaware of what an RRID is, an identifier used to register lab materials and software, let alone where to register or find associated RRIDs for these materials using the SciCrunch database, which catalogs all registered RRIDs. For an overview of key resources that ASAP commonly uses, see Table 4. Adding to the confusion are the requests made by software developers to cite a specific publication describing the software versus an RRID or DOI number linking to software directly, which can generate confusion about which best practice to follow. Moreover, if the preexisting software being used has not already been registered with a permanent identifier in the SciCrunch database or other repositories such as Zenodo, there is also a hesitancy to register the software on someone else’s behalf. Moreover, doing so may also create multiple RRIDs for the same software instance. For example, we observed instances where the same software package may have multiple RRIDs associated with it, and it isn’t clear which RRID to select for citation purposes.

**Table 4:** This table is a quick lookup table for all of the resources utilized in either generating the manuscript or referenced as an open science tool within the text. Key resources.

Version	Software name	URL	Description of how ASAP uses this tool	RRID
n/a	OA.Report	https://oa.report/	Tool used to identify manuscripts citing funder affiliation in acknowledgments	RRID:SCR_023288
n/a	GitHub	https://github.com/	Web-hosting service for software development projects. Researchers can share their developed code here.	RRID:SCR_002630
n/a	SciCrunch	https://scicrunch.org/	Centralized catalog to look up registered RRIDs for lab materials and software tools. Researchers can use this tool to identify how to register different lab material outputs.	RRID:SCR_003115
n/a	Zenodo	https://zenodo.org/	All-purpose data repository that can also share preserved GitHub code.	RRID:SCR_004129
n/a	Protocols.io	http://protocols.io/	Platform to upload and share recipe-style protocols. Researchers can register their protocols referenced in methods sections of paper here.	RRID:SCR_010490
n/a	Cellosaurus	https://web.expasy.org/cellosaurus/	Database of immortalized cell lines used in biomedical research. Researchers can register newly created cell lines here.	RRID:SCR_013869
n/a	Addgene	http://www.addgene.org/	Plasmid repository facilitating the archiving and distributing of DNA-based reagents and associated data. Researchers can register their plasmids here.	RRID:SCR_002037
3.4.0	ggplot2	https://ggplot2.tidyverse.org	Open-source software package for statistical programming language R used for data visualization.	RRID:SCR_014601
4.1.1	R Project for Statistical Computing	https://www.r-project.org/	Software environment and programming language for statistical computing and graphics.	RRID:SCR_001905
v0.0.1	DataSeer	https://dataseer.ai/	Platform to generate reports from manuscript that identify and assess research outputs in a manuscript.	RRID:SCR_023027

### Lab resources

The greatest challenge for authors was registering new lab materials generated from the manuscript. Even in the second version of manuscripts, only 6% of newly generated materials had an RRID associated with the output (Fig 2E). This is due to 2 main issues. First, getting a resource deposited and available for distribution in a registry takes time and money. Some of this can be allayed through preregistration mechanisms that assign an RRID to the material prior to it being publicly available. Certain resource types already have a preregistration workflow in place (e.g., a cell line can be preregistered with an RRID through Cellosaurus, a database of immortalized cell lines used in biomedical research), but there is a substantial knowledge gap within the researcher community about these workflows. Second, there needs to be more clarity on how an RRID should be registered, as different agencies govern different resource types with different procedures (e.g., antibodies are handled separately from cell lines which are handled separately from plasmids). Another source of confusion is in how to handle specific stable resources that currently do not have registering bodies that can mint RRIDs, such as newly generated gene probes or compounds generated for research purposes. There is no clear framework for using patent numbers or other isolated identifiers in such use cases. This results in a complex and fragmented landscape that is confusing for the average researcher to navigate and for the funder to provide clear guidelines.

### Protocols

There was a strong upward trend toward sharing protocols (Fig 2G and 2H and Table 3). The percentage shared jumped from 13% in the first version to 53% in the second version on average. During our outreach, we learned that the most significant barrier to sharing methods was that authors were worried about plagiarism and didn’t understand why we required the methods sections to link to a recipe-style registered protocol. Most believed that the description in the methods section of a manuscript was enough information. To help teams, ASAP provided information on how protocols are not copyrighted material, emphasizing that credit should still be given, but anyone can upload a protocol (if it was not a trademarked secret) regardless of who generated it. Additionally, ASAP staff shared lessons learned from the Cancer Reproducibility Project, which was a great motivator in increasing adoption rate of sharing recipe-style protocols and registering with platforms like protocols.io [20].

### Other considerations

In our experience, even with PM support, the ones responsible for depositing the data are trainees with little to no formal education in their graduate school career about key considerations for curating datasets and other research outputs. To help train our network, ASAP recently developed a checklist for repository deposition [21], explaining the components to consider and the rationale for why it matters. In future, as open science policies evolve and become more widespread, educational training should become a required component of future research program curriculums.

### Roadmap for the future

From this compliance assessment, we identified 2 main barriers to compliance. First, there is a lack of clarity on how research outputs should be registered, deposited, and cited. Second, little to no educational training is provided on the current best open science practices and how they should be implemented. To address these barriers, we posit that a community framework should be developed for sharing research outputs, along with a concerted effort to educate the research community on best practices for implementation. Along those lines, a few initiatives are cropping up to help train the research community, such as Code Ocean focused on best practices for code sharing, FASEB (Federation of American Societies for Experimental Biology) DataWorks Help Desk which provides resources relate to the development of data management plans, Open Data Institute which works with various stakeholders to establish best data practices, and the Open Research Funders Group (ORFG) Open & Equitable Program which has resources for funders to consider around open science implementation. We encourage these initiatives to collectively centralize their resources, creating a common guideline shared across all initiatives, making it easier for others to leverage their offerings.

Our initial focus has been on ensuring that research outputs are being appropriately cited in research manuscripts. Although this is the first and necessary step towards ensuring that outputs are findable, it does not necessarily mean that the research output being linked is reusable. Work predominantly done in studying reproducibility in the psychology field has demonstrated that curating datasets and code are critical to ensuring reusability and many fall short in doing the proper curation when uploading their outputs [22–26]. To address these concerns, we are developing reporting standards based on output type through various working groups within our grantee network. A true definition of open science success, which can only be tested a few years down the line, is when others can utilize our datasets for meta-analysis and/or training validation sets to test hypothesis and when others are using the lab materials, protocols, and code developed by our community in their own experiments.

While it is expected that open science standards may change in the coming years as the landscape evolves, it will also be essential to note the current best practices for a particular point in time to ensure a consistent message and framework upon which compliance monitoring can be built. ASAP aims to contribute to the open science community by educating our growing network (currently over 1,000+ individuals). Our PMs have monthly training sessions to stay current with ASAP requirements and best practices in open science as well as share roadblocks with the ASAP open science staff. Our goal is for the PMs to serve as open science ambassadors for their respective CRN teams.

We recognize that another source of friction arising in the open science community is understanding whose responsibility is it to ensure open science practices are followed. Should accountability lie with the funders that pay for the research to be done, the publishers who disseminate the research findings, or the academic institutions that provide the oversight and facilities in which the research is conducted? Our belief is that the responsibility does not lie with one sole organization. Rather, the entire research ecosystem needs to take collective action. As more funding bodies and institutions embrace open research, there are 7 vital actions that, if taken collectively, would ignite rapid culture change and assist in compliance with the emerging landscape of open research goals and policies:

1. **Align policies and offer direct incentives** for collaboration, transparency, and reproducibility in research communication.
2. **Define compliance and establish open standards** for tracking and measuring open research practices so that it is clear when compliance is reached.
3. **Establish common best practices and standards,** including repository use, appropriate persistent identifiers (PIDs) to use depending on research output type, and clear instructions on how to share and log outputs efficiently.
4. **Invest in infrastructure** that helps existing repositories become FAIR compliant, creates pathways for appropriate PID assignment, removes PID delays, and standardizes compliance metrics.
5. **Normalize the workflow for sharing and reporting on outputs**, an extensible publicly owned research output management schema should be created and used across all infrastructures to prevent the fractured metadata landscape that plagues the published record today.
6. **Automate and streamline sharing** by depositing article supplementary materials into FAIR repositories with PIDs assigned, detecting datasets that haven’t been shared, linking deposits to ORCIDs and articles, and updating outputs based on connected publications.
7. **Pool these actions** across funding bodies, institutions, and publishers so that they can scale.

Our analysis shows that most authors are willing to comply with open science practices if the policies and requirements are clearly outlined and education and support is provided through the PM role to assist with open science compliance—ideally, a PM with experience utilizing the datasets generated by the grantee. By working with other funders, institutions, and research communities, ASAP hopes to help influence the widespread uptake of collaborative and open research practices and contribute to a shared knowledge base on establishing this as the norm for the coming years.
